# Validating and comparing the CAM-ICU and the ICDSC in mild and moderate traumatic brain injury patients: a multicenter prospective study

**DOI:** 10.1186/cc13665

**Published:** 2014-03-17

**Authors:** E Bebawi, L Deslauriers, AA Tessier, AJ Frenette, MM Perreault, MS Delisle, JC Bertrand, M Desjardins, F Bernard, P Rico, K Khwaja, L Burry, DR Williamson

**Affiliations:** 1 Hôpital du Sacré-Coeur de Montreal, Canada; 2 McGill University Health Center, Montreal, Canada; 3 Mount Sinai Hospital, Toronto, Canada

## Introduction

The Confusion Assessment Method for the ICU (CAM- ICU) and the Intensive Care Delirium Screening Checklist (ICDSC) are recommended for routine delirium screening. However, literature about delirium assessment in traumatic brain injury (TBI) remains scarce. The aim of our study was to evaluate the validity and reliability of the CAM-ICU and the ICDSC for delirium assessment in patients with mild to moderate TBI.

## Methods

A prospective observational study of mild to moderate TBI adult patients in two critical care trauma centers. Patients underwent delirium assessment on days 3, 5 and 7 with the CAM-ICU and the ICDSC. Psychiatrists or neurointensivists evaluated delirium using the DSM-IV criteria for delirium. Assessments results were blinded and performed independently. Criterion validity of the CAM-ICU and the ICDSC were calculated from 2 × 2 frequency tables using standard definitions of sensitivity, specificity, positive and negative predictive value, and overall accuracy. Because delirium was assessed repeatedly, estimates of 95% CIs for binary repeated data using generalized estimating equation in conjunction with the Huber-White estimator were performed. Inter-rater reliability for the CAM-ICU and ICDSC was assessed with the kappa coefficient.

## Results

During an 8-month period, 61 patients (mean age 56.4 ± 18.5 years, mean APACHE II score 11.4 ± 6.5, mean GCS 13 ± 2) were enrolled. The overall sensitivity and specificity for CAM-ICU (62% and 74%, respectively) and ICDSC (64% and 79%, respectively) were similar. The overall kappa for inter-rater reliability for the CAM-ICU and ICDSC was 0.64 and 0.68, respectively. In subgroup analyses, the CAM- ICU and ICDSC showed increased sensitivity and decreased specificity in moderate TBI as well as deeper levels of sedation (RASS -2 to -3).

## Conclusion

Criterion validity and inter-rater reliability of the CAM-ICU and ICDSC were good. Severity of TBI and depth of sedation influence delirium assessments. Clinicians should be aware of those limitations before using these clinical tools in this population.

